# Effects of different levels of chili straw supplementation on growth performance, rumen fungal community structure, function and economic benefits in sheep

**DOI:** 10.3389/fmicb.2025.1585992

**Published:** 2025-04-29

**Authors:** Jinlong Li, Congbin Xu, Linjiao He, Yong Tuo, Yuxia Yang, Yan Ma, Tongjun Guo

**Affiliations:** ^1^Feed Research Institute of Xinjiang Academy of Animal Husbandry Sciences, Urumqi, China; ^2^Xinjiang Key Laboratory of Herbivorous Livestock Feed Biotechnology, Urumqi, China; ^3^College of Animal Science, Xinjiang Agricultural University, Urumqi, China

**Keywords:** chili straw, sheep, growth performance, rumen fungi, economic benefits

## Abstract

Chili straw is a crop residue that can be used as an unconventional feed additive in animal production, exhibiting potential value in improving animal health. This study investigated the effects of dietary chili straw on growth performance, rumen fungal community composition, and metabolic function in sheep. Thirty finishing sheep (3-4 months old) with similar body weights were randomly divided into three groups (*n* = 10) and fed diets containing 0, 10, and 20% capsicum straw (CS) for 63 days, including a 7-day adaptation period. At the end of the trial, body weights were recorded, and rumen fluid samples were collected to assess growth performance, fungal diversity, and functional profiles. Dry matter intake (DMI) significantly increased in the 10% CS group (*P* < 0.01), which was 9.71% higher than that of CON group, while DMI and final body weight of 20% CS group (*P* < 0.01 or *P* < 0.05) decreased by 6.81 and 8.81%, respectively, compared with CON group. Final body weight and average daily gain (ADG) showed an upward trend in the 10% CS group (*P* > 0.05), while ADG displayed a downward trend in the 20% CS group (*P* > 0.05). The ACE and Chao1 indices were significantly elevated in the 20% CS group (*P* < 0.05). Principal coordinate analysis (PCoA) and non-metric multidimensional scaling analysis (NMDS) showed that compared with the control (CON) group, the representative points of the 20% CS group gathered more closely. Relative abundances of Ascomycota and Cladosporium increased, whereas Basidiomycota and Kazachstania decreased in CS-supplemented groups (*P* > 0.05). FUNGuild functional prediction indicated increased relative abundances of symbiotrophic and pathotrophic fungi and decreased saprotrophic fungi in CS groups (*P* > 0.05). The gross profit and net profit of the CS10% group were significantly higher than those of the other groups, which were 15.16 and 24.44% higher than those of the control group, respectively. Thus, adding 10% CS to sheep feed can improve the composition of rumen fungi and growth performance, thereby increasing profitability in sheep production.

## 1 Introduction

Chili pepper (*Capsicum annuum* L.), a herbaceous annual plant in the Solanaceae family, is native to South America and has been widely cultivated across tropical and temperate regions globally (Carrizo et al., 2016; Liu et al., 2023). FAO statistical data indicate that global chili production exceeds 40 million tons annually (Food and Agriculture Organization of the United Nations).^1^ While generating significant economic value, this crop also yields a by-product called chili straw (CS) that makes up 30-40% of the total biomass. Traditionally, such agricultural waste has been incinerated or disposed of in a landfill, which not only wastes resources, but also releases greenhouse gases like CO_2_ and methane (Li et al., 2018). In recent years, with the advancement of global carbon neutrality strategies, the utilization of agricultural by-products as feed resources has become a major focus in animal husbandry research. CS contains 92.02% dry matter (DM), 6.81% crude protein (CP), 1.13% ether extract (EE), and 54.66% neutral detergent fiber (NDF). Its crude protein content (6.81%) is higher than that of corn stover (5.00%) and wheat straw (3.00%), while its neutral detergent fiber (54.66%) is lower than corn stover (70.00%) and wheat straw (81.00%), demonstrating potential value as ruminant roughage (NRC, 2007; Li J. L. et al., 2024). However, the physical barriers formed by its acid detergent fiber (45.00%) and lignin (38.00%) may constrain the bioaccessibility of its nutrients, highlighting the need to determine how well it can be digested by rumen microbiota.

The unique rumen ecosystem of ruminants harbors a diverse microbial community comprising bacteria, archaea, protozoa, and fungi. Although rumen fungi constitute only 5-20% of the rumen microbiota, they play a pivotal role in fiber degradation. They mitigate the negative effects of lignin in the rumen while generating fermentable sugars (Anil et al., 2015). Studies have shown that under sterile conditions, rumen fungi can degrade more than half of the DM in feed (Fliegerova et al., 2015). Rumen fungi deploy a specialized rhizoidal system to mechanically disrupt lignocellulose, which is then degraded by secreted fiber-hydrolyzing cellulases and ligninases that enzymatically break down the structural polysaccharides in feed. This process creates favorable conditions for subsequent bacterial fermentation (Hess et al., 2020; Dos Santos et al., 2021; Wei et al., 2022). Notably, fungal taxa like Ascomycota, Neocallimastigomycota, and Neocallimastix exhibit substrate specificity, exclusively targeting fibrous materials. This functional differentiation suggests that dietary adjustments could enhance overall digestive efficiency by modulating fungal community composition and activity.

Recent reports have shown that CS added to feed can enhance animal production performance, energy and protein metabolism, improve the rumen microenvironment, and promote livestock health and product quality. Research has demonstrated that dietary supplementation with 10-15% CS increases serum glucose, total protein, and albumin levels in sheep, effectively enhancing energy and protein metabolism (Li J. L. et al., 2024). CS contains bioactive compounds such as flavonoids, polyphenols, and polysaccharides, which are critical for reducing systemic inflammation and improving animal health. Xu et al. (2024) reported that feeding CS to Ipora meat rabbits elevated serum IgA and IgM levels, thereby enhancing immune function. Lu (2023) observed that CS supplementation in sheep increased the abundance of Firmicutes in the rumen, optimizing the microbial ecosystem. CS inclusion elevated the relative abundance of the *Rikenellaceae_RC9_gut_*group, a family of fiber-degrading rumen bacteria, reduced saturated fatty acid (SFA) content in lamb meat from 57.22 to 53.30%, and increased the unsaturated-to-saturated fatty acid ratio from 0.08 to 0.10 (Li J. et al., 2024). This study hypothesized that these mechanisms may also involve fungal-mediated enhancement of fiber degradation. However, existing research has focused predominantly on rumen bacterial communities, with limited exploration of fungal community dynamics and functional roles.

Building on prior research, this study aimed to investigate the effects of dietary supplementation with CS on growth performance and rumen fungal diversity in finishing-phase sheep. By analyzing differences in the rumen fungal population, this research seeks to further elucidate the mechanisms underlying the effect of dietary CS on sheep, to provide a reference for the use of CS as an alternative feed resource in sheep production.

## 2 Materials and methods

The protocols for animal care and handling in this study were designed in compliance with the Guidelines for the Care and Use of Laboratory Animals in China and approved by the Animal Care Committee of Xinjiang Academy of Animal Sciences, China (Protocol License No. 3 20230507, Urumqi, China).

### 2.1 Materials

Chili straw is composed of the air-dried roots and stems remaining after the fruit of Tianjiao Hongguan chili [registration no. GPD chili (2017) 650019] was harvested. The chili straw used in this study was provided by Xinjiang Yuanda Green Agriculture Development Co. Ltd. (Kashgar, China). This study used LC-MS to detect bioactive substances in CS, and a total of 1,189 bioactive substances were found. The nutritional level and active substance composition of chili straw have been reported in our previous study (Li J. et al., 2024).

### 2.2 Experimental design, animal diets, and management

This study is part of a series of experiments on using CS as a feed supplement in meat sheep production. The procedures were performed at the sheep farm of Xinjiang Taihe Agriculture and Animal Husbandry Technology Co., Ltd. (Bachu County, Kashgar Prefecture, Xinjiang Uygur Autonomous Region, China; longitude 78.61143; latitude 39.779815). In this study, 30 F1 hybrid lambs from a cross of Dorper × Hu sheep were used, 3–4 months old, with body weights of 29.53 ± 2.07 kg. The experimental animals were randomly divided into three groups (*n* = 10). Sheep in the control group (CON) were fed the standard total mixed granule diet, and those in the test group (CS) received the total mixed granule diet containing 10% and 20% air-dried CS. The energy level and protein content of the CON and CS diets were the same. The experiment lasted for 63 days, with the first 7 days being used as the diet adaptation period, and the following 56 days for the feeding experiment. The basal diet contained the nutrients recommended by the NRC (2007) for sheep (Table 1). The CON and CS groups were raised under the same conditions, and each group of 10 sheep was kept in a single enclosure. Before the experiment began, the animals were dewormed and the feeding area was thoroughly disinfected. The animals were fed at 8:00 and 19:30 every day, and had free access to food and water. During the test, the feeding environment was dry and well-ventilated. The daily feeding amount was adjusted according to the previous day’s intake to maintain 2-4% residual feed.

**TABLE 1:** Composition and nutrient levels of the basal diet (DM basis, %).

Item	Control	CS 10%	CS 20%
**Ingredients**			
Alfalfa hay	10.88	7.54	5.22
Soybean straw	14.03	10.45	6.80
Straw	18.12	14.49	10.22
Chili straw	0.00	10.00	20.00
Corn	31.04	30.75	30.48
Wheat bran	7.89	7.74	7.67
Cottonseed meal	8.88	10.29	11.35
Sunflower meal	4.98	4.57	4.09
Limestone	0.12	0.12	0.11
NaCl	0.53	0.53	0.53
CaHPO_4_	0.51	0.50	0.51
NaHCO_3_	0.51	0.51	0.51
Premix^1^	2.51	2.51	2.51
Total	100.00	100.00	100.00
**Nutritional level^2^**			
GE/(MJ/kg)	15.80	15.69	15.57
CP	13.11	13.46	13.86
EE	2.12	1.97	2.45
NDF	55.50	54.06	54.81
ADF	25.15	24.56	28.52
Ca	1.35	1.79	1.63
P	0.47	0.31	0.42

^1^The premix contained the following per kg of ration: VitA 150,000 IU, VitD3 56,500 IU, Vit E 8000 IU, Se (Na_2_SeO_3_) 14 mg, I (KI) 58 mg, Cu (CuSO_4_) 290 mg, Mn (MnSO_4_) 1925 mg, Zn (ZnO) 2050 mg, and Co (CoSO_4_) 24 mg.

^2^Nutrient contents were measured values. NaCl, sodium chloride; CaHPO_4_, calcium hydrogen phosphate; NaHCO_3_, sodium bicarbonate; GE, total energy; CP, crude protein; EE, ether extract; NDF, neutral detergent fiber; ADF, acid detergent fiber; Ca, calcium; P, phosphorus.

### 2.3 Sample collections

On day 57, four experimental sheep were selected from each group based on body weight and euthanized by exsanguination through the jugular artery and vein following electric stunning after 12 h of fasting and 2 h of water deprivation as required by the animal welfare protocol. Rumen fluid was collected from the upper opening of the rumen and stored at –80°C.

### 2.4 Indicator measurements

#### 2.4.1 Growth performance

The feed amount and feed residue were weighed and recorded daily, DM intake (DMI) was calculated, and the sheep were weighed on an empty stomach before morning feeding on the first and 57th day of the official period, and the average daily gain (ADG) and ratio of food consumed to weight gained (F/G) were calculated (Zhang et al., 2024).

DMI (kg/d) = (amount of DM in feed per group per day - amount of DM in residual feed per group per day)/number of experimental sheep per group

ADG (g/d) = (final weight of each sheep - initial weight of each sheep)/experimental days

F/G = DMI/ADG

#### 2.4.2 DNA extraction, PCR, rumen fungi sequencing

DNA was extracted from rumen fluid using the TGuide S96 DNA kit (Tiangen Biotech Co., Ltd., Beijing, China). PCR was performed with the universal primer set, ITS1F: 5′-CTTGGTCATTTAGAGGAAGTAA-3′ and ITS2R: 5′-GCTGCGTTCTTCATCGATGC-3′. The PCR reaction mix was prepared according to the method of Niu et al. (2023). The PCR reactions contained KOD FX Neo 0.2 μL and ddH_2_O to 10 μL and VnF and Vn R were selected according to the amplification region. The PCR reactions were conducted using the following program: 3 min of denaturation at 95°C; 27 cycles of 30 s at 95°C; 30 s for annealing at 55°C; 45 s for elongation at 72°C; and a final extension at 72°C for 10 min. The ITS gene was detected and analyzed using the approach established by Tong et al. (2023). PCR amplicons were purified with Agencourt AMPure XP Beads (Beckman Coulter, Indianapolis, IN) and quantified with the Qubit dsDNA HS assay kit and Qubit 4.0 fluorometer (Invitrogen, Thermo Fisher Scientific, Oregon, United States). Libraries were constructed from equal amounts of pooled amplicons and sequencing was performed on an Illumina Novaseq 6000 platform (Illumina, San Diego, CA, United States).

### 2.5 Statistical analysis

This study organized the collected experimental data in Microsoft Excel 2021, and used SPSS26.0 statistical software (version 26.0; IBM, Armonk, NY, United States) to perform one-way analysis of variance (ANOVA) on the collated data, followed by multiple comparisons using the Duncan *post-hoc* method. The results of the analyzed data were presented as the mean, and the standard error (SEM) was used to represent the degree of variation in each group, with *P* < 0.05 considered significant. This study used UNITE as the fungal reference database and a naive Bayesian classifier to annotate sequences to obtain corresponding species classification information. QIIME software was used to analyze beta diversity of rumen fungi, and the Bray-Curtis algorithm was selected to calculate separation distances. QIIME software was also used to analyze the relative abundance of different species at various taxonomic levels. Alpha index plots, species composition plots, and correlation heat maps were generated using Origin (version 2024, OriginLab, Hampton, United States) software, and correlation analyses were performed using Pearson’s algorithm.

## 3 Results

### 3.1 Growth performance

Compared with the CON group and the CS20% group, the DMI of the CS10% group was significantly increased by 9.71 and 17.79%, respectively. Compared with the CON group, the DMI of the CS20% group was significantly reduced by 6.81% (Table 2). The final body weight (FBW) of the CS20% group was significantly lower than that of the CON group and the CS10% group by 8.81% (*P* < 0.05) and 12.18% (*P* < 0.01), respectively. The ADG of the CS10 % group was significantly increased by 47.80% compared with the CS20 % group (*P* < 0.01). In addition, with the increase of the addition level of chili straw in the diet, the DMI and FBW of sheep showed linear and quadratic changes (*P* < 0.05 or *P* < 0.01), and the ADG showed quadratic changes (*P* < 0.05). There was no significant difference in other indicators among the groups (*P* > 0.05).

**TABLE 2:** Effects of different levels of chili straw on the growth performance of sheep.

Items	Control	CS10%	CS20%	SEM	*P*-value		
					**Trt**	**L**	**Q**
Dry matter intake (DMI, kg/d)	1.75^Bb^	1.92^Aa^	1.63^Cc^	0.013	<0.001	0.002	<0.001
Initial body weight (IBW, kg)	29.93	29.43	29.23	0.399	0.776	0.494	0.865
Final body weight (FBW, kg)	44.52^ABa^	46.23^Aa^	40.60^Bb^	0.759	0.004	0.017	0.011
Average daily weight gain (ADG, g/d)	260.52^ABab^	300.00^Aa^	202.98^Bb^	13.835	0.010	0.060	0.012
Feed conversion ratio (F/G)	6.98	6.85	8.61	0.415	0.157	0.109	0.227

CS10%, 10% chili straw group; CS20%, 20% chili straw group. SEM is the pooled standard error between three groups, and *P*-values show significance. Different superscripts show significant differences within a row:^A, B, C^ (*P* < 0.01), ^a, b, c^ (*P* < 0.05).

### 3.2 Effects of CS on abundance, diversity, and composition of rumen fungi

#### 3.2.1 Venn diagram of overlapping rumen fungi

**FIGURE 1:**
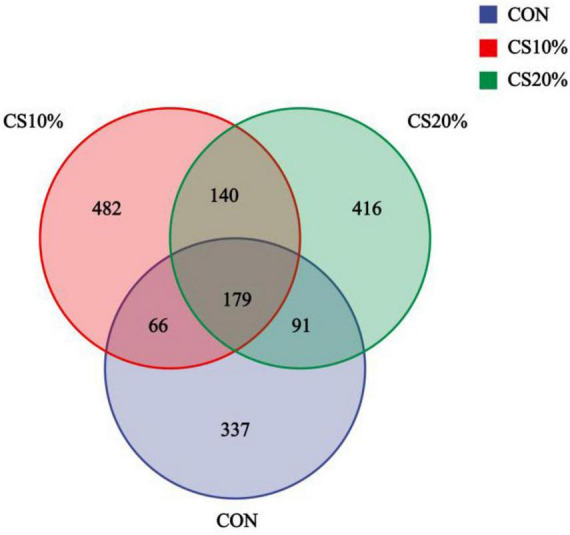
Venn diagram of ruminal fungi.

The coverage rates for the sample sequences exceeded 99% for all groups, indicating that the samples correctly reflected the profiles in the rumen fungal population. The sequence data from the 12 samples yielded a total of 920,010 paired reads. After QC and assembly, 825,931 high-quality clean reads were generated. Each sample produced ≥ 46,957 clean reads, with an average of 68,827. The sequence clusters had 97% similarity, resulting in 1,711 OTUs (operational taxonomic units) in 12 samples (Figure 1). A total of 179 OTUs were shared between samples from different groups, of which, the CON, CS10%, and CS20% had 337, 482, and 416 unique OTUs, respectively.

#### 3.2.2 Alpha diversity analyses of rumen fungi

**FIGURE 2:**
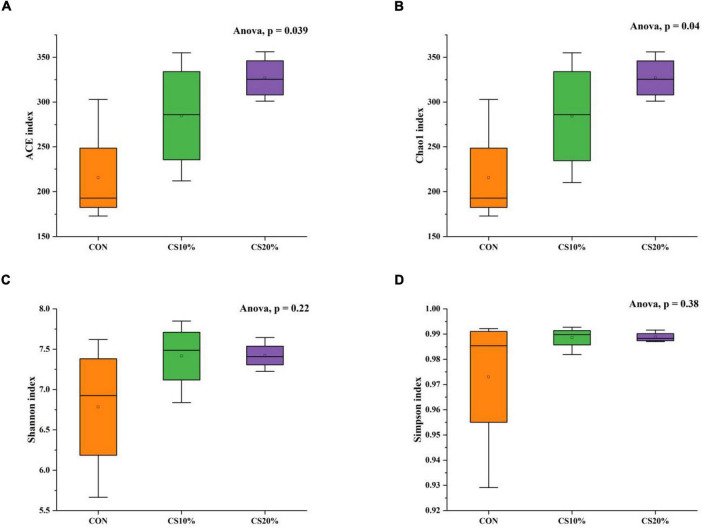
Alpha diversity indices of rumen fungi from the three groups. **(A)** ACE index of species richness; **(B)** chao1 index of species richness; **(C)** shannon index of species diversity; **(D)** simpson index of species diversity.

The alpha diversity determination of the rumen fungi (Figures 2A, B) shows that the ACE index and Chao1 index in the CS20% group were significantly higher than those in the CON group (*P* < 0.05), but comparison with the other groups revealed no significant differences (*P* > 0.05). From Figures 2C, D, there was no significant difference in Shannon index and Simpson index between CS group and CON group (*P* > 0.05).

#### 3.2.3 Beta diversity analyses of rumen fungi

**FIGURE 3:**
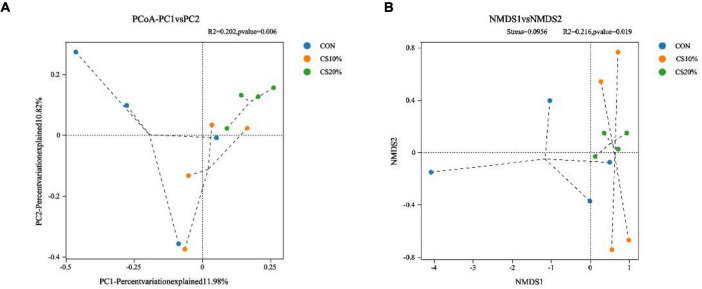
Beta diversity analysis of rumen fungi. **(A)** PCoA principal axis analysis; **(B)** NMDS non-metric multidimensional scaling analysis.

The PCoA and NMDS calculations utilizing the Bray-Curtis distance matrix (Figures 3A, B) show that with increasing percentage of CS, the points representing the additive groups were closely clustered, and more separated from the CON group than from each other. The representative points of the CS20% group and the CON group could be effectively distinguished (PCoA, *P* = 0.006; NMDS, *P* = 0.019).

#### 3.2.4 Determination of the composition and abundance of ruminal fungi

**FIGURE 4:**
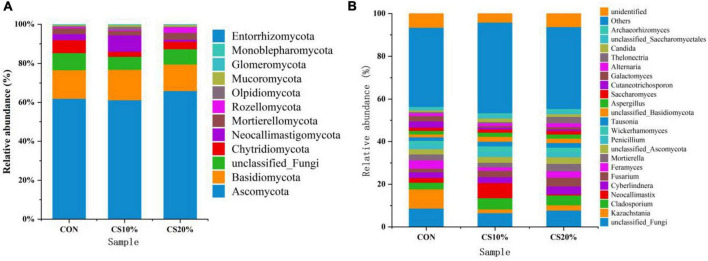
Percent distribution of fungal phyla **(A)** and genera **(B)** in different groups.

The structure of the rumen fungal community is depicted in Figures 4A, B. Taxonomic annotation of representative sequences was achieved with a simple Bayesian classifier, identifying 12 fungal phyla and 24 genera with a relative abundance exceeding 1% from 12 samples of gastrointestinal contents from sheep. The dominant phyla included *Ascomycota* (63.00%), *Basidiomycota* (15.00%), *Chytridiomycota* (4.67%), and *Neocallimastigomycota* (4.00%), which together accounted for ∼95% of the total sequences. Compared with CON, CS20% had a higher relative abundance of Ascomycota, though this difference wasn’t significant (*P* > 0.05).

At the genus level, *Kazachstania*, *Cladosporium*, and *Neocallimastix* were dominant, with relative abundances of 4.33, 4.33, and 3.00%, respectively. Compared with CON, the CS group showed a lower relative abundance of *Kazachstania* and higher relative abundances of *Cladosporium* and *Fusarium*, though none of these differences reached statistical significance (*P* > 0.05).

#### 3.2.5 Prediction of function in the rumen fungal community

**FIGURE 5:**
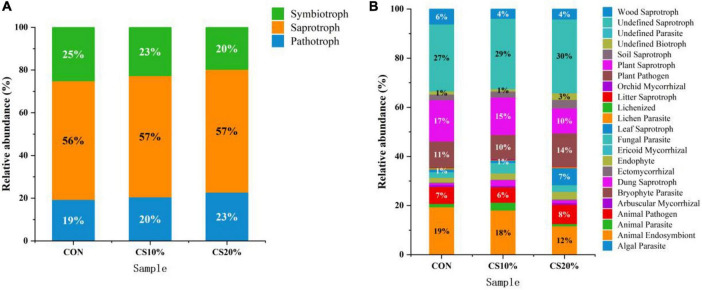
Predicted abundance plots of rumen fungi from different treatment groups with specific FUNGuild functions. **(A)** Trophic classification; **(B)** FUNGuild classification.

This study predicted the functional profiles of rumen fungi in each experimental group using the FUNGuild database. The results revealed that rumen fungi could be classified into three trophic modes based on nutritional strategies: pathotrophic, symbiotrophic, and saprotrophic, with saprotrophic fungi dominating at 56.67%. Compared to CON, the CS group exhibited a higher relative abundance of pathotrophic fungi and a lower relative abundance of symbiotrophic fungi, but these differences were non-significant (*P* > 0.05) (Figure 5A).

The symbiotic community encompassed 20 ecological functional groups. The pathotrophic-symbiotrophic-saprotrophic fungi primarily included animal pathogens, animal endosymbionts, and dung saprotrophs. Notably, the relative abundance of these functional groups in the CS20% group (30.00%) was lower than that in the CON group (43.00%) (Figure 5B).

#### 3.2.6 Relationship between growth performance, ruminal fermentation, and rumen fungi

**FIGURE 6:**
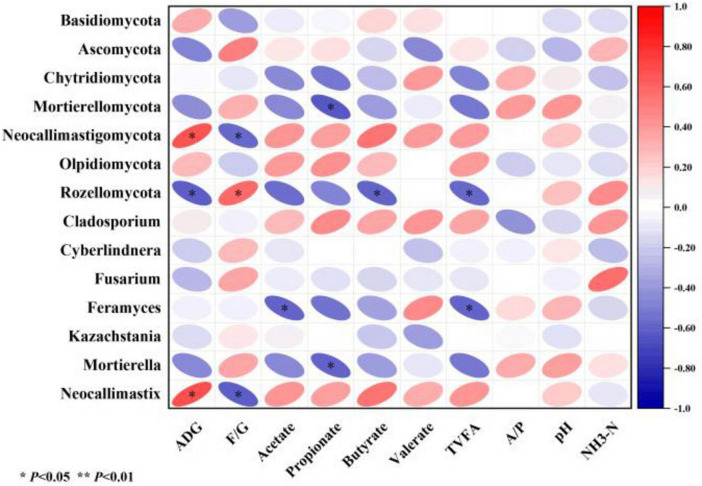
Heatmap depicting the relationships among relative fungal abundance, rumen fermentation parameters, and growth performance. *P < 0.05, **P < 0.01. ADG, avg daily gain; F/G, feed to gain ratio; A/P, acetate/propionate.

This study used Pearson’s algorithm to calculate correlation coefficients and constructed a correlation heatmap to illustrate the relationships among growth performance, ruminal fermentation, and rumen fungi. ADG showed a significantly positive correlation with the abundance of *Neocallimastigomycota* and *Neocallimastix* in the rumen (*P* < 0.05), and a significantly negative correlation with *Rozellomycota* (*P* < 0.05). The feed-to-gain ratio (F/G) exhibited a significantly negative correlation with *Neocallimastigomycota* and *Neocallimastix* (*P* < 0.05), but a significantly positive correlation with *Rozellomycota* (*P* < 0.05). Acetate concentration in the rumen was negatively correlated with *Feramyces* (*P* < 0.05), while propionate showed a negative correlation with *Mortierellomycota* and *Mortierella* (*P* < 0.05). Butyrate was negatively correlated with *Rozellomycota* (*P* < 0.05), and total volatile FAs demonstrated negative correlations with both *Rozellomycota* and Feramyces (*P* < 0.05) (Figure 6).

### 3.3 Effect of percent content of chili straw on sheep economic benefits

This study observed that the cost per kg of feed for each group was 2.07-2.24 yuan, and the unit price decreased with increasing CS percentage. The gross profit of the CS10% group increased by 15.16% compared with the CON group (*P* < 0.05), and the net profit increased by 24.44% (*P* > 0.05). In addition, with the increase of chili straw addition level, gross profit and net profit showed a quadratic change (*P* < 0.05) (Table 3).

**TABLE 3:** Results of feeding different CS percentages on sheep economic benefits.

Item	Control	CS10%	CS20%	SEM	*P*-value		
					**Trt**	**L**	**Q**
Feed unit price (yuan/kg)	2.24	2.15	2.07	–	–	–	–
ADFI	1.75*^Bb^*	1.92*^Aa^*	1.63*^Cc^*	0.013	<0.001	<0.001	<0.001
Feed cost	219.52	231.17	188.95	–	–	–	–
Sheep live weight unit price	31	31	31	–	–	–	–
Total weight gain, kg	14.59*^Aa^*	16.80*^ABab^*	11.37*^Bb^*	0.775	0.010	0.060	0.012
Gain from weight gain	452.26*^Aa^*	520.80*^ABab^*	352.37*^Bb^*	24.017	0.010	0.060	0.012
Net profit	232.74	289.63	163.42	22.255	0.062	0.183	0.047

CS, chili straw. SEM is pooled standard error. *P* < 0.05 is significant. Different superscripts within row = significantly different means: ^A,B,C^ (*P* < 0.01), ^a,b,c^ (*P* < 0.05).

## 4 Discussion

Chili straw (CS) contains a certain amount of capsaicin, which stimulates feed intake in ruminants. Increased feed intake enhances energy and protein uptake, thereby promoting animal growth and development. Su et al. (2023) reported that supplementing diets with 0.15 or 0.30 mL/d of capsaicin improved dry matter intake (DMI) in calves but had no significant effect on ADG. Cheng Z. Z. et al. (2023) found that feeding Suffolk sheep a total mixed ration containing 15% CS fermented with *Lactobacillus plantarum* did not negatively affect final body weight or ADG. Fu et al. (2024) reported that adding 11.25-45.00% CS to beef cattle diets increased final weight, ADG, and DMI at inclusion levels of 11.25-33.75%, but ADG and DMI declined when the inclusion level reached 45%. In this study, the CS10% group exhibited significant increases in DMI and ADG, while the CS20% group showed reductions in both metrics. The improvements in the CS10% group may be attributed to capsaicin-stimulated feed intake and enhanced nutrient uptake. Additionally, bioactive compounds in CS likely modulated rumen microbiota structure and improved fermentation efficiency, thereby facilitating nutrient digestion and absorption. However, the decline in DMI and ADG observed in the CS20% group may result from excessive capsaicin content impairing feed palatability (Ricci et al., 2021), aligning with findings by Su et al. (2023), Cheng J. F. et al. (2023), and Fu et al. (2024). The discrepancy between our results and those of Cheng Z. Z. et al. (2023) in Suffolk sheep could stem from two factors. Fermentation may reduce capsaicin levels in CS, diminishing its stimulatory effect on intake; and the use of uniformly pelleted feed in this study ensured better feed homogeneity and intake consistency compared to a total mixed ration. These findings suggest that chili pepper straw, as an unconventional feed resource, can enhance growth performance in sheep when incorporated into diets. However, to optimize outcomes, CS inclusion levels should be carefully controlled, with 10% identified as the optimal threshold.

The diverse microbial community in the rumen plays a pivotal role in ruminant growth. Studies indicated that the composition of rumen microbiota significantly influences ruminant growth and health (Li et al., 2019; Loh et al., 2020). Further research has demonstrated that fibrous feeds from different sources markedly affect rumen microbial diversity in ruminants (Wang et al., 2023). Fiber intake enhances dietary microbial diversity (Fernandes et al., 2014). In this study, CS was added to the diet of sheep, and the rumen fluid was collected for fungal ITS sequencing. The results revealed elevated ACE and Chao1 indices in CS-supplemented groups, with the CS20% group showing significant increases. PCoA and NMDS analyses based on the Bray-Curtis distance matrix demonstrated distinct separation between the CS and CON groups, with tighter clustering of CS group samples. These findings collectively indicate that CS supplementation modulates fungal richness in the rumen.

The fungal diversity at the phylum level in rumen samples of all sheep groups was consistent. Regardless of the CON or CS groups, Ascomycota and Basidiomycota dominated, collectively accounting for over 75% of rumen fungi, aligning with findings in other ruminants (Liggenstoffer et al., 2010; Wu et al., 2023). Ascomycota primarily degrade organic substrates such as lignin, keratin, and plant cellulose, while Basidiomycota specialize in lignocellulose breakdown (Xing et al., 2020). In this study, the higher relative abundances of Ascomycota and Basidiomycota in CS groups may stem from two factors: the elevated neutral detergent fiber (NDF) content in CS diets stimulating their growth, and the inherently higher abundance of these phyla in the fungi carried by the ingested CS (Chen et al., 2024; Jose et al., 2024). Neocallimastigomycota emerged as a subdominant phylum in the rumen of sheep. This anaerobic fungal group specializes in cellulose degradation (Cheng et al., 2018) and provides nutrients to ruminants by metabolizing rumen-degradable proteins (Belanche et al., 2012). Neocallimastigomycota exhibited a significant positive correlation with ADG. Its relative abundance was significantly higher in the CS10% group compared to CON, being consistent with the increased apparent digestibility of crude protein observed in prior studies (Li J. L. et al., 2024). Conversely, the CS20% group showed reduced *Neocallimastigomycota* abundance, likely due to substrate limitations—moderate cellulose levels stimulate growth, while excessive cellulose may overwhelm enzymatic capacity, hindering proliferation. These findings suggest that care must be taken to be sure the appropriate percentage of CS is added to the feed to enhance Neocallimastigomycota proliferation, thereby promoting ruminal fiber and protein degradation, boosting volatile fatty acid (VFA) production, and ultimately improving energy supply and growth performance in ruminants.

The dominant fungal genera identified in this study included *Kazachstania*, *Cladosporium*, and *Neocallimastix*. *Kazachstania*, a member of the Basidiomycota, is primarily involved in carbohydrate metabolism in feed (Jood et al., 2017). Zhang et al. (2019) demonstrated that elevated fiber levels reduce the abundance of *Kazachstania* in the rumen of Tan sheep under grazing and confined feeding being consistent, consistent with our findings. The declining trend of *Kazachstania* with increasing CS inclusion may stem from the higher lignification and fiber content of CS, which suppresses its proliferation. *Cladosporium*, a member of the Ascomycota, participates in cellulose degradation by secreting cellulases to break down structural carbohydrates in feed (Andlar et al., 2018). The increasing abundance of *Cladosporium* with higher CS inclusion may be attributed to the elevated neutral detergent fiber (NDF) content in CS, which stimulates its enzymatic activity. *Neocallimastix*, classified under Neocallimastigomycota, shares functional similarities with its parent phylum, producing cellulolytic enzymes that digest cellulose and lignin in roughage (Dollhofer et al., 2018). It also contributes to protein and amino acid metabolism in the rumen (Belanche et al., 2012). *Neocallimastix* exhibited a significant positive correlation with ADG. The higher relative abundance of *Neocallimastix* in the CS10% group compared to CON aligns with previous findings on improved crude protein digestibility (Li J. L. et al., 2024). However, a substantial proportion of fungal genes remained unannotated due to limited fungal genomic data in public databases such as NCBI, KEGG, and Swiss-Prot (Solomon et al., 2016), highlighting the need for expanded fungal genome resources to advance functional analyses.

The metabolic function and wellbeing of ruminants are linked to the composition of rumen microbiota. Using FUNGuild software, this study predicted the ecological functions of rumen fungi. During feed intake and digestion in ruminants, lignin content in feed not only affects palatability but also restricts bacterial degradation of cellulose (Cao et al., 2016), but fungal lignin- and cellulose-degrading capabilities are resistant to this inhibition (Sigoillot et al., 2012). Rumen fungi are categorized into three ecological functional groups (pathotrophic, symbiotrophic, and saprotrophic) which decompose organic matter through parasitism, symbiosis, and saprotrophy, respectively. The ecological functions of rumen fungi are tightly coupled with their community structure (Moraïs and Mizrahi, 2019). In this study, symbiotrophic fungi dominated the rumen fungal community of sheep. Ascomycota, the predominant phylum in the sheep rumen, is primarily comprised of symbiotrophic fungi that degrade organic substrates (Xing et al., 2020). The CS group exhibited a decreasing trend in the relative abundance of saprotrophic fungi, likely due to the higher lignocellulose content in CS diets, which limited substrate availability for enzyme secretion and thus suppressed saprotroph proliferation. Additionally, the CS group showed a reduced trend in animal endosymbionts, being consistent with the decline in *Kazachstania* abundance. A potential limitation of this study is that only rumen liquid-associated fungi were analyzed, whereas solid-adherent fungi were not assessed, which may explain the lack of pronounced effects of CS inclusion on fungal community composition.

Economic benefits are closely related to sheep growth performance and the price of feed raw materials (Tekletsadik et al., 2013). In this study, adding 10% CS to the feed reduced the unit price of the feed. This indicates that the application of CS in ruminant diets is a potential means to reduce costs and improve growth performance. However, as the percentage of CS in the diet increased, the total weight gain of sheep in the CS group first increased and then decreased. The optimal CS level was 10%, resulting in an increase in total weight gain of 15.15% relative to CON, and an increase of 24.44% in the net profit. Mohammed et al. (2021) and Hou et al. (2025) added tomato pomace and black wolfberry leaves to broiler and sheep diets, respectively, increased daily weight gain and gross profit. In this study, the use of CS to feed sheep also improved economic benefits, which indicated that feed rich in functional substances could improve the efficiency of animal husbandry production.

## 5 Conclusion

The present findings indicated that the addition of 10% CS to the diet can enhance growth performance and the profitability of meat sheep farming, but 20% CS was inhibitory. Under the experimental conditions of this study, dietary supplementation with 10% CS improved the structural composition of rumen fungi and significantly increased the DMI and ADG of sheep, thereby improving production performance and economic benefits. These findings provide practical guidance for the utilization of CS in sheep production and offer theoretical insights into its role in improving rumen health. In the future, it is necessary to analyze the dynamic balance mechanism of CS active substances in ‘Rumen microbial-host metabolic axis’ through multi-omics joint analysis, so as to provide a theoretical tool for precision animal husbandry.

## Data Availability

The rumen fungal data were deposited into the National Center for Biotechnology Information (NCBI) Sequence Read Archive (SRA) with the accession numbers RPJNA1226559. The other original data were uploaded to Figshare: https://figshare.com/s/a910e357c2d5a7aac80e.

## Footnotes

1
https://www.fao.org/home/en/

## References

[B1] Andlar M. Rezić T. Marğetko N. Kracher D. Ludwig R. Šantek B. (2018). Lignocellulose degradation: An overview of fungi and fungal enzymes involved in lignocellulose degradation. *Eng. Life Sci.* 18 768–778. 10.1002/elsc.201800039 32624871 PMC6999254

[B2] Anil K. P. Rameshwar S. Devki N. K. (2015). *Rumen Microbiology: From Evolution to Revolution.* New Delhi: Springer.

[B3] Belanche A. Doreau M. Edwards J. E. Moorby J. M. Pinloche E. Newbold C. J. (2012). Shifts in the rumen microbiota due to the type of carbohydrate and level of protein ingested by dairy cattle are associated with changes in rumen fermentation. *J. Nutr.* 142 1684–1692. 10.3945/jn.112.159574 22833657

[B4] Cao B. B. Jin X. Yang H. J. Li S. L. Jiang L. S. (2016). Microbial release of ferulic and p-coumaric acids from forages and their digestibility in lactating cows fed total mixed rations with different forage combinations. *J. Sci. Food Agric.* 96 650–655. 10.1002/jsfa.7136 25675865

[B5] Carrizo G. C. Barfuss M. H. Sehr E. M. Barboza G. E. Samuel R. Moscone E. A. (2016). Phylogenetic relationships, diversification and expansion of chili peppers (Capsicum, Solanaceae). *Ann. Bot.* 118 35–51. 10.1093/aob/mcw079 27245634 PMC4934398

[B6] Chen Z. Wang W. Chen L. Zhang P. Liu Z. Yang X. (2024). Effects of pepper-maize intercropping on the physicochemical properties, microbial communities, and metabolites of rhizosphere and bulk soils. *Environ. Microbiome* 19:108. 10.1186/s40793-024-00653-7 39696399 PMC11657000

[B7] Cheng J. F. Xie C. Q. Xu X. Zhou X. Ding L. H. Zhou W. D. (2023). Effects of pepper straw powder on growth performance, nutrient digestibility and skin quality of rex rabbits. *J. Zhejiang Agric. Sci.* 64 2544–2548. 10.16178/j.issn.0528-9017.20230695

[B8] Cheng Y. Shi Q. Sun R. Liang D. Li Y. Li Y. (2018). The biotechnological potential of anaerobic fungi on fiber degradation and methane production. *World J. Microbiol. Biotechnol.* 34:155. 10.1007/s11274-018-2539-z 30276481

[B9] Cheng Z. Z. Aibibula Y. Li F. M. Yisilayi D. Wan J. C. (2023). Evaluation of feeding effect of pepper straw and its silage with different treatments on lambs. *Chin. J. Anim. Nutr.* 36 387–396. 10.12418/CJAN2024.035

[B10] Dollhofer V. Dandikas V. Dorn-In S. Bauer C. Lebuhn M. Bauer J. (2018). Accelerated biogas production from lignocellulosic biomass after pre-treatment with Neocallimastix frontalis. *Bioresour. Technol.* 264 219–227. 10.1016/j.biortech.2018.05.068 29807329

[B11] Dos Santos T. A. X. Fernandes L. M. G. Carvalho P. P. X. Júnior V. S. M. Fonseca S. A. Chaves A. S. (2021). Performance and microbiota of the digestive tract of Nellore calves supplemented with fungi isolated from bovine rumen. *Vet. World* 14 2686–2693. 10.14202/vetworld.2021.2686-2693 34903926 PMC8654770

[B12] Fernandes K. A. Kittelmann S. Rogers C. W. Gee E. K. Bolwell C. F. Bermingham E. N. (2014). Faecal microbiota of forage-fed horses in New Zealand and the population dynamics of microbial communities following dietary change. *PLoS One* 9:e112846. 10.1371/journal.pone.0112846 25383707 PMC4226576

[B13] Fliegerova K. Kaerger K. Kirk P. Voigt K. (2015). *Rumen Microbiology: From Evolution to Revolution: Rumen Fungi.* New Delhi: Springer India, 97–112. 10.1007/978-81-322-2401-3_7

[B14] Fu H. Xia Z. Zhang X. Tian H. (2024). Effects of different proportions of chili straw silage as a replacement for corn straw on growth performance, nutrient digestibility, and serum biochemical indices of beef cattle. *China Feed* 35 181–185. 10.15906/j.cnki.cn11-2975/s.2023060043-06

[B15] Hess M. Paul S. S. Puniya A. K. Van Der Giezen M. Shaw C. Edwards J. E. (2020). Anaerobic fungi: Past, present, and future. *Front. Microbiol.* 11:584893. 10.3389/fmicb.2020.584893 33193229 PMC7609409

[B16] Hou L. Duan P. Yang Y. Shah A. Li J. Xu C. (2025). Effects of residual black wolfberry fruit on growth performance, rumen fermentation parameters, microflora and economic benefits of fattening sheep. *Front. Vet. Sci*. 11:1528126. 10.3389/fvets.2024.1528126 39867601 PMC11760609

[B17] Jood I. Hoff J. W. Setati M. E. (2017). Evaluating fermentation characteristics of Kazachstania spp. and their potential influence on wine quality. *World J. Microbiol. Biotechnol.* 33:129. 10.1007/s11274-017-2299-1 28585169

[B18] Jose S. Malla M. A. Renuka N. Bux F. Kumari S. (2024). Cyanobacteria-green microalgae consortia enhance soil fertility and plant growth by shaping the native soil microbiome of Capsicum annuum. *Rhizosphere* 30:100892. 10.1016/j.rhisph.2024.100892

[B19] Li F. Li C. Chen Y. Liu J. Zhang C. Irving B. (2019). Host genetics influence the rumen microbiota and heritable rumen microbial features associate with feed efficiency in cattle. *Microbiome* 7:92. 10.1186/s40168-019-0699-1 31196178 PMC6567441

[B20] Li H. Dai M. Dai S. Dong X. (2018). Current status and environment impact of direct straw return in China’s cropland - A review. *Ecotoxicol. Environ. Saf.* 159 293–300. 10.1016/j.ecoenv.2018.05.014 29763811

[B21] Li J. L. Guo T. J. Zang C. J. Zhang Z. J. Tuo Y. (2024). Effects of different pepper stalk level diets on growth performance, nutrient apparent digestibility and serum indices of dorper×hu hybrid lambs. *Chin. J. Anim. Nutr.* 36 4531–4543. 10.12418/CJAN2024.389

[B22] Li J. Tuo Y. He L. Ma Y. Zhang Z. Cheng Z. (2024). Effects of chili straw on rumen fermentation, meat quality, amino acid and fatty acid contents, and rumen bacteria diversity in sheep. *Front. Microbiol.* 15:1525612. 10.3389/fmicb.2024.1525612 39877758 PMC11773153

[B23] Liggenstoffer A. S. Youssef N. H. Couger M. B. Elshahed M. S. (2010). Phylogenetic diversity and community structure of anaerobic gut fungi (phylum Neocallimastigomycota) in ruminant and non-ruminant herbivores. *Isme J.* 4 1225–1235. 10.1038/ismej.2010.49 20410935

[B24] Liu F. Zhao J. Sun H. Xiong C. Sun X. Wang X. (2023). Genomes of cultivated and wild Capsicum species provide insights into pepper domestication and population differentiation. *Nat. Commun.* 14:5487. 10.1038/s41467-023-41251-4 37679363 PMC10484947

[B25] Loh Z. H. Ouwerkerk D. Klieve A. V. Hungerford N. L. Fletcher M. T. (2020). Toxin degradation by rumen microorganisms: A review. *Toxins* 12:64. 10.3390/toxins12100664 33092236 PMC7590051

[B26] Lu Y. M. (2023). *Effects of Capsicum Straw Micro-Storage on Rumen Fermentationparameters, Gastrointestinal Morphology, Microflora and Metabolite of Mutton Sheep.* Master Master: Shihezi University.

[B27] Mohammed L. Sallam E. Edris S. Khalifa O. Soliman M. Shehata S. (2021). Growth performance, economic efficiency, meat quality, and gene expression in two broiler breeds fed different levels of tomato pomace. *Vet. Res. Commun*. 45 381–397. 10.1007/s11259-021-09819-x 34458932

[B28] Moraïs S. Mizrahi I. (2019). The road not taken: The rumen microbiome, functional groups, and community states. *Trends Microbiol.* 27 538–549. 10.1016/j.tim.2018.12.011 30679075

[B29] Niu J. Liu X. Xu J. Li F. Wang J. Zhang X. (2023). Effects of silage diet on meat quality through shaping gut microbiota in finishing pigs. *Microbiol. Spectrum* 11 2416–2422. 10.1128/spectrum.02416-22 36507700 PMC9927310

[B30] NRC. (2007). *Nutrient Requirements of Small Ruminants: Sheep, Goats, Cervids, and New World Camelids.* Washington, DC: National Academy Press.

[B31] Ricci S. Rivera-Chacon R. Petri R. M. Sener-Aydemir A. Sharma S. Reisinger N. (2021). Supplementation with phytogenic compounds modulates salivation and salivary physico-chemical composition in cattle fed a high-concentrate diet. *Front. Physiol.* 12:645529. 10.3389/fphys.2021.645529 34149443 PMC8209472

[B32] Sigoillot J.-C. Berrin J.-G. Bey M. Lesage-Meessen L. Levasseur A. Lomascolo A. (2012). “Fungal strategies for lignin degradation,” in *Advances in Botanical Research*, eds Jouanin L. Lapierre C. (Cambridge, MA: Academic Press), 263–308.

[B33] Solomon K. V. Haitjema C. H. Henske J. K. Gilmore S. P. Borges-Rivera D. Lipzen A. (2016). Early-branching gut fungi possess a large, comprehensive array of biomass-degrading enzymes. *Science* 351 1192–1195. 10.1126/science.aad1431 26912365 PMC5098331

[B34] Su M. She Y. Deng M. Guo Y. Li Y. Liu G. (2023). The effect of capsaicin on growth performance, antioxidant capacity, immunity and gut micro-organisms of calves. *Animals (Basel)* 13:2309. 10.3390/ani13142309 37508086 PMC10376287

[B35] Tekletsadik E. Solomon M. Mengistu U. (2013). The effect of barley bran, linseed mealand their mixes supplementation on the performances, carcass characteristics andeconomic return of arsi-bale sheep. *Small. Rumin. Res.* 114 35–40. 10.1016/j.smallrumres.2013.05.010

[B36] Tong Y. Wu J. Guo W. Yang Z. Wang H. Liu H. (2023). The effect of combining millet and corn straw as source forage for beef cattle diets on ruminal degradability and fungal community. *Animals (Basel)* 13:548. 10.3390/ani13040548 36830335 PMC9951761

[B37] Wang X. Shi B. Zuo Z. Qi Y. Zhao S. Zhang X. (2023). Effects of two different straw pellets on yak growth performance and ruminal microbiota during cold season. *Animals (Basel)* 13:548. 10.3390/ani13030335 36766224 PMC9913257

[B38] Wei Y. Yang H. Wang Z. Zhao J. Qi H. Wang C. (2022). Roughage biodegradation by natural co-cultures of rumen fungi and methanogens from Qinghai yaks. *AMB Express* 12:123. 10.1186/s13568-022-01462-2 36121525 PMC9485394

[B39] Wu Y. Jiao C. Diao Q. Tu Y. (2023). Effect of dietary and age changes on ruminal microbial diversity in holstein calves. *Microorganisms* 12:1331. 10.3390/microorganisms12010012 38276181 PMC10818949

[B40] Xing B. S. Han Y. Wang X. C. Wen J. Cao S. Zhang K. (2020). Persistent action of cow rumen microorganisms in enhancing biodegradation of wheat straw by rumen fermentation. *Sci. Total Environ.* 715:136529. 10.1016/j.scitotenv.2020.136529 32007902

[B41] Xu H. Nie F. Huang A. Hou J. Zhao J. Hastong L. (2024). Effect of chili pepper straw on growth performance, blood biochemical and immunological indices of hyplus meat rabbits. *Chin. Feed Ind.* 45, 135–139. 10.13302/j.cnki.fi.2024.13.020

[B42] Zhang J. Li N. Guo T. Hu D. Xu X. Zhang L. (2019). Effects of different feeding methods on rumen fungus flora of Tan sheep by high-throughput sequencing of ITS rDNA. *J. Gansu Agric. Univer.* 54 25–34. 10.13432/j.cnki.jgsau.2019.05.004

[B43] Zhang W. Ren F. Zang C. Yang F. Li X. Huang X. (2024). Effects of dietary addition of ellagic acid on rumen metabolism, nutrient apparent digestibility, and growth performance in Kazakh sheep. *Front. Vet. Sci.* 11:13. 10.3389/fvets.2024.1334026 38379922 PMC10877003

